# Review on Resistive Switching Devices Based on Multiferroic BiFeO_3_

**DOI:** 10.3390/nano13081325

**Published:** 2023-04-10

**Authors:** Xianyue Zhao, Stephan Menzel, Ilia Polian, Heidemarie Schmidt, Nan Du

**Affiliations:** 1 Institute for Solid State Physics, Friedrich Schiller University Jena, Helmholtzweg 3, 07743 Jena, Germany; xianyue.zhao@uni-jena.de (X.Z.);; 2 Department of Quantum Detection, Leibniz Institute of Photonic Technology (IPHT), Albert-Einstein-Str. 9, 07745 Jena, Germany; 3 Peter Grünberg Institut (PGI-7), Forschungszentrum Juelich GmbH, Wilhelm-Johnen-Str., 52428 Juelich, Germany; 4 Institute of Computer Science and Computer Engineering, University of Stuttgart, Pfaffenwaldring 47, 70569 Stuttgart, Germany

**Keywords:** multiferroic BiFeO_3_, resistive switching, crystal structure, ferroelectricity, FTJs, VCM, switching property, energy consumption

## Abstract

This review provides a comprehensive examination of the state-of-the-art research on resistive switching (RS) in BiFeO_3_ (BFO)-based memristive devices. By exploring possible fabrication techniques for preparing the functional BFO layers in memristive devices, the constructed lattice systems and corresponding crystal types responsible for RS behaviors in BFO-based memristive devices are analyzed. The physical mechanisms underlying RS in BFO-based memristive devices, i.e., ferroelectricity and valence change memory, are thoroughly reviewed, and the impact of various effects such as the doping effect, especially in the BFO layer, is evaluated. Finally, this review provides the applications of BFO devices and discusses the valid criteria for evaluating the energy consumption in RS and potential optimization techniques for memristive devices.

## 1. Introduction

Memristive devices have garnered significant attention from numerous research groups in the realm of memory and computing over the past decade, as they present a promising possibility to go beyond von Neumann computing, owing to their non-volatile reconfigurable resistive state [1,2]. Multiferroic nanomaterials have also received great interest due to their unique combination of magnetic and ferroelectric properties, which can be utilized for a variety of applications, including resistive switching (RS). Multiferroic materials possess both ferroelectricity and magnetism at room temperature, providing unique advantages with both electrical and magnetic degrees of freedom, thereby delivering higher performance and more functions to RS devices. The RS devices based on different metal/insulator/metal (MIM) exhibit RS behaviors, e.g., Ni-Zn-Co ferrite (NZFO) [3] and YMn$O_{3}$ (YMO) [4]. BiFe$O_{3}$ (BFO) [5,6,7] is one such multiferroic material that has been shown to exhibit RS behavior, making it a promising candidate for the development of memristive devices. However, BFO has certain advantages over these materials, particularly in terms of its ferroelectric and magnetic properties, such as polarization, coercive field, remanent magnetization, and Curie temperature. For instance, BFO has a higher polarization and a lower coercive field than NZFO [3], and it has a higher Curie temperature and a lower remanent magnetization than YMO [8,9]. However, the RS mechanism responsible for the RS behavior in BFO memristive devices is still unclear, although several models have been proposed to understand the RS behavior, such as conductive filament [10], Schottky barrier [11], and ferroelectric tunneling [12]. The proposed mechanism models for BFO-based memristive devices represent reasonable assumptions that can be validated for specific devices, and it is up to individual researchers to demonstrate which mechanism their device belongs to through experiments and analysis. The goal of this review is to provide a summary of the current understanding of the complex switching mechanism and RS behavior of BFO-based memristive devices and to explore their interrelationships to offer valuable insights for improving the practical performance of these devices.

**Figure 1:**
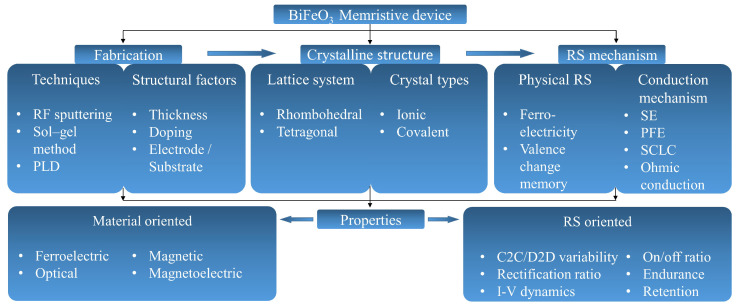
Schematic diagram of a BFO memristive device, briefly illustrating the key aspects of its common fabrication techniques, crystalline structures, RS mechanism, and various properties. (SE: Schottky emission, PFE: Poole–Frenkel emission, SCLC: space–charge-limited conduction, C2C: cycle to cycle, D2D: device to device).

Figure 1 illustrates the common fabrication techniques, crystalline structures, RS mechanisms, and key properties of BFO layers and constructed BFO-based memristive devices, highlighting the intricate interplay between crystalline structure, device fabrication technique, and RS behavior. The fabrication technique affects the quality and composition of the crystalline structure, which in turn determines the RS mechanism and properties of the device. Understanding these relationships is essential for optimizing the performance and functionality of the BFO memristive device for potential applications in nonvolatile memory and neuromorphic computing.

This review work comprehensively investigates RS in BFO layers and discusses the potential application of this material in memristive devices. Section 2 examines various fabrication techniques for creating BFO layers, including the sol–gel method and pulsed laser deposition (PLD), along with their parameters. Section 3 analyzes the crystal structures of BFO, including its ferroelectric and conductive properties, and considers the factors that influence the material’s crystal structure. Section 4 examines the RS mechanisms of BFO memristive devices, specifically focusing on ferroelectricity and valence change memory (VCM). Finally, in Section 5, RS properties of BFO memristive device and their applications of BFO devices are discussed, with particular emphasis on the energy consumption of memristive devices and potential optimization techniques for memristive devices.

## 2. Fabrication Techniques

The preparation of high-quality memristive devices involves the utilization of various deposition techniques, which can be broadly categorized into physical and chemical deposition methods. Notable physical deposition techniques include PLD, radio frequency sputtering (RF Sputtering), and molecular beam epitaxy (MBE). On the other hand, common chemical deposition techniques encompass the sol–gel method, chemical vapor deposition (CVD), and atomic layer deposition (ALD). Chemical deposition technology, such as the sol–gel method, is commonly applied as it is an economical, convenient, and effective method for preparing BFO-based memristive devices [10,13,14]. The key parameters of the sol–gel method are precursor concentration, deposition temperature, deposition time, cleaning steps, and annealing conditions. The sol–gel method can produce high-quality BFO films with uniformity and has the possibility of coating on substrates of any size and large area [15].

**Figure 2:**
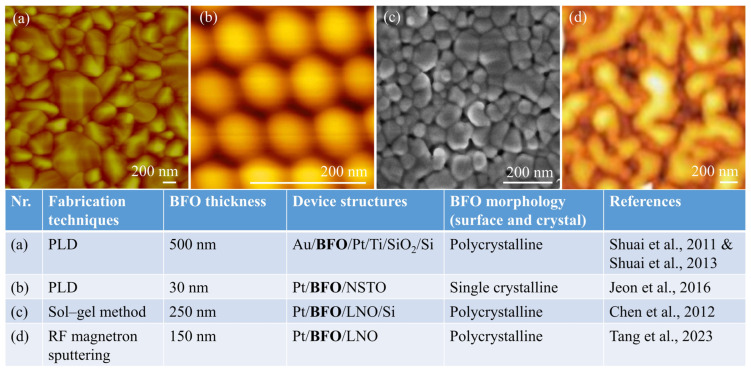
Crystal structures of BFO memristive devices summarized in the table along with atomic force microscope topography images demonstrating various fabrication techniques, (**a**) Thin film, (**b**) Nanoislands, (**c**) Thin film, and (**d**) Thin film. All images are reproduced with permission from the corresponding references in Refs. [7,17,24,25,26].

As a kind of physical deposition technology, PLD is commonly used in the preparation of metal oxide layers, including BFO memristive devices [16]. The fabrication parameters for BFO memristive devices using PLD are highly variable and are contingent upon the specific research or fabrication process. Typical parameters for fabricating BFO memristive devices by PLD include laser energy density of 1–2 $J/{\mathrm{cm}}^{2}$, laser repetition rate of 2–10 Hz, substrate temperature of 450–800 °C, oxygen pressure of 10–120 mTorr, and target-substrate distance of 3–6 cm [5,17,18,19,20,21]. For example, higher laser repetition rates during deposition can affect the RS behavior of BFO memristive devices by changing the thickness and quality of the BFO film, resulting in thicker films and a higher on/off ratio [21]. Lamichhane et al. increased the on/off ratio and reduced the leakage current of a BFO memristive device by increasing the laser energy density [22]. Furthermore, other factors, including substrate material, doping, and the presence of buffer layers, can also influence the properties of the BFO memristive device. Additionally, PLD offers several advantages over other physical deposition systems because of its fast deposition time and compatibility with oxygen and other inert gases [23]. Figure 2 summarizes several representative morphologies to discuss the primary fabrication techniques utilized for BFO memristive devices. The PLD technique offers an advantage for growing BFO nanoislands and improving the growth of single crystals.

The selection of the PLD-deposited dielectric RS layers, i.e., BFO layer and their physical feature size can significantly influence the RS dynamics in BFO memristive devices. The thickness of the dielectric layer is one of the most critical parameters. Even by using the same fabricating technique of a single device, various factors, particularly changes in fabricating parameters, can lead to different thicknesses and, hence, different RS behaviors. It has been demonstrated that the thickness dependence of the conduction mechanism is linked to oxygen vacancy defects, which decrease gradually with increasing BFO film thickness [27]. The structure of epitaxial BFO films is closely related to the thickness of the film and the epitaxial strain on different substrates. In addition, doping other elements or forming solid solutions can also affect the structure of BFO films. In addition to BFO thin films, BFO nanoislands are also a research hotspot. The morphology and size of BFO microstructures largely affect their properties.

High-quality single-crystal BFO nanoislands can be fabricated by PLD with thinner thickness and higher density than BFO films. This is of great significance to the application of the BFO memristive device crossbar in the future. In this review, we have focused on the influence of fabrication technique changes on the performance of memristive devices. However, the effects of electrode/substrate materials and doping factors cannot be ignored. We have provided several examples to separately introduce these two factors.

While fabricating BFO thin films, the epitaxial strain applied by the substrate may cause the compound to assume a reduced symmetry structure [28]. The wide range of possible crystal structures highlights the strong influence of the substrate on the structural properties of the thin films and thus also on the properties and behavior of BFO devices. In the choice of substrate, different types of contacts can be created using metal oxide substrate materials with different work functions and carrier concentrations. Understanding the impact of different interfacial contacts on the device performance has great implications for the device application. Metal oxide materials, such as LaNi$O_{3}$ (LNO) and SrRu$O_{3}$ (SRO), are now widely used as BFO memristive device substrates to improve the quality of BFO. For example, in studied BFO polycrystalline films, the film orientation and ferroelectric properties can be controlled by different substrate layers. Yan et al. [29] reported on the deposition of BFO thin films on LNO and SRO-coated silicon substrates by PLD and found that the BFO film deposited on the LNO-coated Si substrate shows a (001) preferred orientation and higher ferroelectric properties, while the BFO film grown on the SRO-buffered Si substrate shows a random orientation.

Elemental doping is considered another potential method to improve the electrical and ferroelectric properties of BFO films. BFO as a perovskite is a class of materials with the general formula ABX${}_{3}$, in which A is the larger cation, B is the smaller cation, and X is the anion. Studies have shown that the oxygen vacancy defect in BFO can be controlled by doping A and B sites, which can effectively reduce the leakage current of BFO thin films [28]. It has been found that replacing Bi${}^{3+}$ at the A site with rare-earth ions such as La${}^{3+}$ [10], Ce${}^{3+}$ [30], Nd${}^{3+}$ [31], Sm${}^{3+}$ [32] and alkaline-earth ions such as Ca${}^{2+}$ [33,34], Sr${}^{2+}$ [35], Ba${}^{3+}$ [18] or replacing Fe${}^{3+}$ at the B site with transition metal ions such as Sc${}^{3+}$ [36], Cr${}^{3+}$ [37], Mn${}^{4+}$ [14], and Ti${}^{4+}$ [38] can significantly affect the physical properties of the resulting materials, including their ferroelectric and piezoelectric behavior, dielectric constant, and conductivity. Makhdoom et al. [39] have provided evidence of a structural phase transition from rhombohedral to pseudo-cubic symmetry in BFO thin films following the addition of 10% Ba. The authors have confirmed the presence of the transition through their comprehensive experimental analysis. In particular, the electrical characterization of the 10% Ba-doped device has revealed a leakage current density that is approximately four orders of magnitude lower than that observed in pure BFO devices.

Furthermore, bilayer or multilayer BFO-based memristive devices are receiving increasing attention for their potential impact on RS behavior. Adding another layer can promote the formation of a vacancy reservoir, and the thickness and composition of the layer can affect the size of the reservoir as well as the migration and formation energies of vacancies [40,41]. Moreover, using multilayer dielectrics can also impact the energy required for the formation and migration of defects, resulting in a decrease in the area of oxygen deficiency [42]. Multiple researchers have studied the impact of bilayer or multilayer dielectrics [43,44,45]. The addition of multiple layers complicates the analysis of their effects on performance. For instance, You et al. [43] reported on the BiFeO${}_{3}$:Ti/BiFeO${}_{3}$ (BFTO/BFO) bilayer structure that exhibits complementary RS behavior distinct from the bipolar RS from a single-layer BFO-based memristive device. The memristive BFO bilayer structure demonstrates unique RS behaviors that enable the realization of compact sequential logic. Another example is the combination of HfO${}_{2}$ and BFO, as reported by Liu et al. [44]. Both HfO${}_{2}$ and BFO can be independently used as memristive device materials in MIM structures. When a 2 nm BFO layer was additionally deposited on the top of HfO${}_{2}$ layer, the performance of the BFO/HfO${}_{2}$-based memristive device is significantly enhanced, achieving a large memory window of 10${}^{4}$ and excellent pulse durability of 10${}^{8}$ cycles in comparison to the HfO${}_{2}$-based memristive device.

In summary, the structure of BFO films can be tailored and the properties of BFO memristive devices can be affected by using fabrication parameters, doping, and the epitaxial strain applied by various substrates as control parameters.

## 3. Crystalline Structures

**Figure 3:**
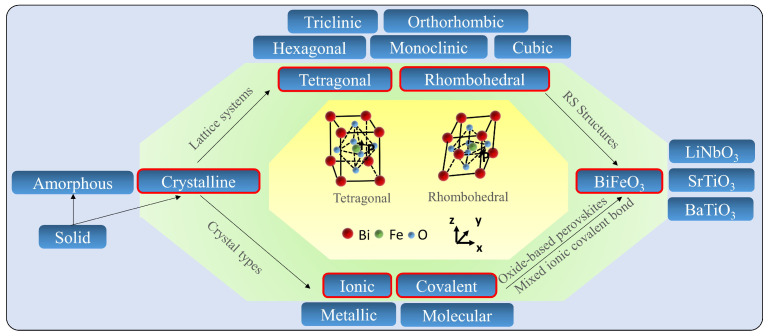
Schematic diagram of the relationship between lattice systems, crystal types, crystalline structures, and memristive devices with a specific focus on crystal symmetry and microstructure of BFO devices.

Solids are characterized by an extended three-dimensional arrangement of atoms, ions, or molecules. When these components are arranged in regularly repeating three-dimensional arrays, solids are classified as solids if they have a long-range order, or they are classified as amorphous if they only have a short-range order. Figure 3 illustrates the interrelationships among the crystalline solids, lattice systems, and crystal types with a specific focus on BFO-based memristive devices with RS behaviors (marked with background color green). It highlights the two lattice structures, i.e., tetragonal and rhombohedral (out of seven existing lattice structures for BFO materials), which can be applied to functional layers for constructing BFO-based memristive devices. Especially, the lattice systems and crystal types, which are relevant for the RS properties of BFO-based memristive devices, are illustrated in comparison to all the available ones of BFO materials (no RS properties so far) existing in the state-of-the-art studies.

Depending on the spatial arrangement of atoms, ions, or molecules in crystalline solids, there are seven types of unit cells in the lattice system: cubic, tetragonal, orthorhombic, monoclinic, hexagonal, rhombohedral, and triclinic. The arrangement of lattices leads to the formation of grains, which can be classified into single crystals with a uniform lattice orientation and no grain boundaries, and polycrystals that consist of multiple crystal grains with different lattice orientations. It is well known that pure BFO typically possesses a rhombohedral crystal structure with a space group of R3c at room temperature. Depending on the type of bonds, crystalline solids are categorized as ionic solids, molecular solids, covalent solids, and metallic solids. Many semiconducting metal oxides, including CaTi$O_{3}$ (CTO) [46], SrTi$O_{3}$ (STO) [47], BFO [48], and BaTi$O_{3}$ (BTO) [49], belong to the class of oxide-based perovskites with ABO${}_{3}$ as their general formula. The chemical bonds in BFO perovskites are considered mixed ionic covalent bonds [50].

The rhombohedral lattice structure of BFO at room temperature is characterized by Fe$O_{6}$ octahedra that are centered and cornered by Fe${}^{3+}$ and Bi${}^{3+}$ ions, respectively. Two perovskite cells are connected along the [111] direction to form a diamond cell. The spontaneous polarization of BFO is due to Bi${}^{3+}$ ion lone pair electrons, Fe${}^{3+}$ displacement polarization, and O${}^{2-}$ electron displacement polarization, which together generate a net electric dipole moment and result in ferroelectricity [51]. Due to the unique positions of Bi${}^{3+}$ and Fe${}^{3+}$ ions in the rhombohedral BFO structure, the substitution of these ions with other elements significantly affects the structure and physical properties of BFO. Moreover, the lattice structure of the substrate affects the crystal structure and properties of BFO thin films due to lattice mismatch between them. Liu et al. [52] used YAl$O_{3}$ substrate for BFO thin films and observed stress and distortion in the BFO thin films due to lattice mismatch. These stresses and distortions have an impact on the electrical, magnetic, and optical properties of BFO thin films. Hence, it is crucial to study the lattice structure of substrates for designing and preparing new materials.

The crystal structure of BFO can be either single crystal or polycrystalline, and it can have two main morphologies: nanoisland or nanothinfilm. Single crystal BFO is easier to form into nanoislands than nanothinfilms due to the special growth process of nanothinfilms. The RS behavior has been observed in BFO with both rhombohedral [25] and tetragonal [5] crystal structures. The rhombohedral lattice is characterized by a three-fold rotational symmetry axis and three equal lattice parameters with a single angle that is not equal to 90 degrees. In BFO, the rhombohedral structure is distorted due to the off-centering of Bi and Fe atoms, resulting in the formation of a permanent polarization. This polarization is responsible for the ferroelectric properties of BFO, which can be manipulated by an external electric field. Therefore, the rhombohedral lattice system is important for the ferroelectric behavior of BFO. However, the multiferroicity properties of BFO are sensitive to fabrication parameters such as annealing or doping, which affect its crystal symmetry and microstructure. Some studies have not solely investigated the memristive behavior of BFO devices but have explored the relationship between ferroelectricity and the crystal structure, which is worthy of comparative analysis. Table 1 provides a comparison of the most prominent crystal structures of BFO devices and their corresponding microstructures. Additionally, ferroelectricity has been demonstrated in the BFO of the tetragonal crystal structures [5]. Another study [24] in the table shows that doping with Ti does not affect the crystal structure but affects the ferroelectricity, and RS behavior still exists. This suggests that ferroelectricity is not the only mechanism responsible for the RS behavior of BFO memristive devices, and other mechanisms must exist to explain it. As detailed in Section 2, the RS behavior of BFO memristive devices is impacted not only by fabrication parameters but also by substrate and doping. These factors often induce changes in the lattice structure, leading to the creation of numerous lattice defects that affect the RS mechanism and RS behavior of BFO memristive devices. Further elaboration on this topic will be provided in Section 4.

The fabrication process of BFO thin films plays a crucial role in determining the crystal structure and orientation of the material, including PLD and sol–gel deposition. These techniques involve depositing thin films of BFO onto a substrate, which can have a significant influence on the crystal structure and orientation of the resulting material. As discussed in Section 2, the choice of substrate can affect the epitaxial relationship between the substrate and the BFO film, which in turn can determine the crystallographic orientation of the film. In addition, the deposition conditions, such as temperature, pressure, and gas composition, can also affect the crystal structure and orientation of the BFO film. Liu et al. [52] conducted a study on the impact of temperature on the crystal structure of BFO thin films. The results showed that the crystal structure changed as indicated by the presence of tetragonal-like structure diffraction peaks from room temperature to 400 °C. Moreover, the c-axis lattice parameter of BFO thin films showed temperature dependence. Specifically, the c-axis parameter underwent elongation and stabilization in the range of 100–250 °C but subsequently decreased as the temperature increased.

## 4. Resistive Switching Mechanisms

In recent years, researchers have noted resistive effects in a range of dielectric materials, emphasizing the vital importance of resistive material selection on the performance of memristive devices. Despite this progress, the details of the physical mechanism of these resistive phenomena are not well understood, and the mechanism of resistive state transition in memristive devices is still a subject of ongoing investigation.

**Figure 4:**
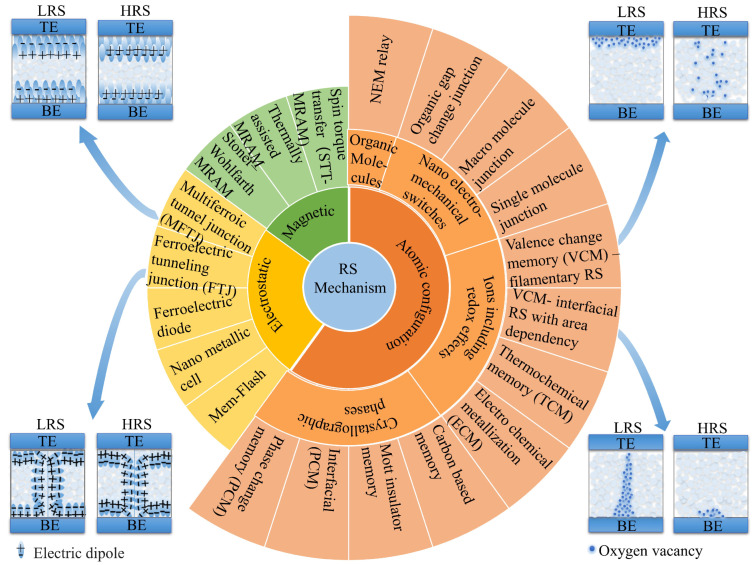
Classification of physical switching mechanisms in memristive devices, with especially demonstrated RS mechanisms in BFO.

Various physical switching mechanisms [54] have been proposed to explain the memristive behaviors of these devices, including magnetic effects, electrostatic effects, and a range of mechanisms based on atomic configurations, as depicted in Figure 4. One of the most extensive groups of physical switching mechanisms leading to memory phenomena is based on various classes of effects based on atomic configuration, which can be subdivided into atomic configurations in ions including redox effects, crystallographic phases, organic molecules, and nanoelectromechanical switches. In the case of BFO-based memristive devices, the dominant physical switching mechanisms can be classified into two categories: ferroelectricity-based electrostatic effect and redox-based on atomic configuration effect, e.g., VCM.

Memristive devices based on the MIM structure exhibit two general types of conduction mechanisms, namely, electrode-limited conduction mechanism and bulk-limited conduction mechanism, depending on the electrical properties at the electrode-switching layer interface and the switching layer itself [55]. The electrode-limited conduction mechanisms include Schottky emission (SE), Fowler–Nordheim tunneling, direct tunneling, and thermionic-field emission. The bulk-limited conduction mechanisms include Poole–Frenkel emission (PFE), hopping conduction, ohmic conduction, space-charge-limited conduction (SCLC), ionic conduction, and grain-boundary-limited conduction. The conduction mechanism in a memristive device can be identified by analyzing the voltage dependence on the current. The electrical characteristics of BFO memristive devices can display non-linear current–voltage behavior in both low-resistance state (LRS) and high-resistance state (HRS). This non-linear behavior indicates the presence of multiple conduction mechanisms contributing to the observed electrical properties. In particular, the non-linear current–voltage response in BFO memristive devices may arise from a combination of ohmic conduction, SE, PFE, and SCLC conduction [56,57,58]. The specific conduction mechanisms governing the observed behavior can be dependent on the unique structural and operational features of the device.

### 4.1. Ferroelectricity in BFO

Ferroelectric materials [59] have garnered significant interest due to their polarization characteristics, which offer advantages such as low energy consumption, high read/write speed, high theoretical storage density, and electromagnetic radiation resistance. As a result, the development and miniaturization of high-density nonvolatile memory devices based on ferroelectric materials have significant implications in the reduced fields of radio frequency systems and space technology [60]. Currently, three main types of devices based on ferroelectric RS behavior have been identified: multiferroelectric tunnel junctions (FMTJ), ferroelectric tunnel junctions (FTJ), and ferroelectric diodes. Materials such as BFO, BTO (BaTiO${}_{3}$, and Pb(ZrTi)$O_{3}$ have been used for FMTJ and MTJ devices, and to enable the growth of ferroelectric materials as heterogeneous epitaxial films on the electrode materials, metal-conducting oxides (e.g., SrRu$O_{3}$, LaNi$O_{3}$), semiconductor oxides (e.g., Nb:SrTi$O_{3}$ (NSTO), ZnO), and magnetic oxides (e.g., La${}_{0.7}$Sr${}_{0.3}$ Mn$O_{3}$, La${}_{0.5}$Sr${}_{0.5}$Co$O_{3}$) are commonly utilized as electrode materials. The combination of these electrodes/substrates with ferroelectric materials leads to diverse interface effects and, subsequently, a range of RS behaviors in different ferroelectric heterostructures. Among the materials exhibiting RS effects, ferroelectric materials have gained substantial attention due to the relationship between their RS behavior and ferroelectric polarization switching. It is well understood that the RS behavior is primarily controlled by polarization. The ferroelectric properties of these materials originate from the polarization of electrons in ferroelectric domains, while magnetic properties are derived from the spin-ordering of electrons in magnetic domains. Thus, the macroscopic properties of multiferroic materials arise from the rotation or migration of microscopic ferroelectric and magnetic domains. As a representative ferroelectric material, BFO has been utilized for constructing memristive devices due to its polarization.

**Figure 5:**
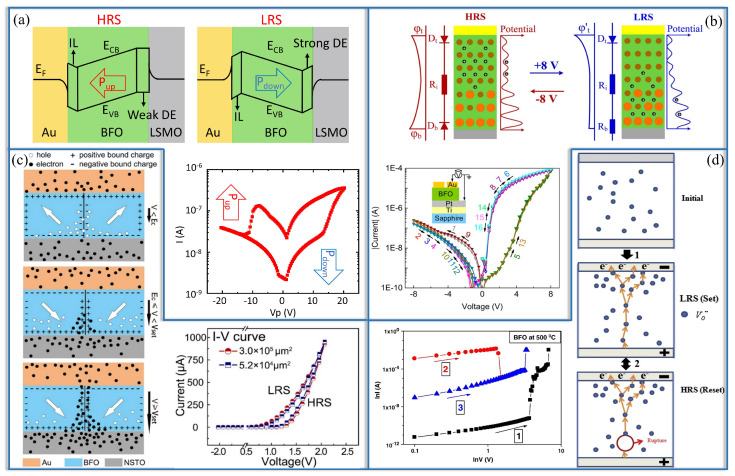
Schematic diagrams of RS mechanisms in memristive devices. (**a**) Multiferroelectric tunnel junction, adapted from Ref. [61], (**b**) Voltage-controlled metal-insulator interface RS, reproduced with permission from Ref. [11], (**c**) Ferroelectric domain wall, reproduced with permission from Ref. [21], and (**d**) Voltage-controlled filament RS, reproduced with permission from Ref. [62]. The switching IV curves are presented as a signature for RS.

As shown in Figure 5a, the investigation by Huang et al. [61] explored the dynamics of ferroelectric domain reversal and its relationship to resistance switching and memristive behaviors in multiferroic heterojunctions based on epitaxial BFO. The study revealed that engineering the domain states could result in achieving multiple and continuously tunable non-volatile resistance states, i.e., memristive states. The BFO-based multiferroic heterojunctions exhibited a resistance switching speed as fast as 30 ns at a write voltage of 20 V. Reducing the thickness of BFO further led to an even faster switching speed (20 ns) and a much lower operation voltage (4 V) in the La${}_{0.6}$Sr${}_{0.4}$MnO${}_{3}$/BFO/La${}_{0.6}$Sr${}_{0.45}$MnO${}_{3}$ MFTJ. Additionally, the MFTJ showed a tunable interfacial magnetoelectric coupling associated with the dynamics of ferroelectric domain switching. These findings highlight the potential of multiferroic heterojunctions based on BFO for achieving fast and tunable memristive devices with interfacial magnetoelectric coupling. Similar to the preparation method of the device in the above work, Han et al. [21] fabricated RS devices based on high-density self-assembled BFO nanoislands grown on an NSTO substrate, as shown in Figure 5c. The formation and rupture of conductive filaments at charged domain walls caused the RS properties due to changes in the charge carrier concentration under an applied voltage. Figure 5c shows a schematic diagram of the charge distribution in the charged domain walls under different applied voltages. The emergence of charged domain walls within BFO nanoislands has been proposed as the conductive path responsible for the observed RS behavior. This phenomenon is attributed to the redistribution of carriers under applied voltages, which is facilitated by the polarization of the domain walls. The presence of polarization helps to establish well-defined RS channels, acting as a bridge between the RS resulting from ferroelectric polarization reversal and that resulting from the motion of Vo${}^{\cdot \cdot }$. *I-V* characteristics reveal distinct RS characteristics with large on/off ratios in the thin films. Therefore, this study provides us with a new approach to improve RS performance by tuning charged domain walls. In fact, since ferroelectric materials contain a large number of polarized ferroelectric domains, the effect of domain walls on nanodevices will inspire new ideas for building nanolevel electronic devices in the future.

### 4.2. Valence Change Memory in BFO

The VCM is a broadly observed switching mechanism in MIM stacks. The switching process of VCM relies on the motion of oxygen vacancies (Vo${}^{\cdot \cdot }$) within the oxide layer and the exchange of Vo${}^{\cdot \cdot }$ with the adjacent electrode/oxide layer [63]. The dielectric layer in these devices can act as a mixed ionic-electronic conductor, and the Vo${}^{\cdot \cdot }$ migrated under the influence of an applied electric field. The used metal oxides in VCM-based MIM memristive devices are HfO${}_{x}$ [64], TaO${}_{x}$ [65], BFO [11], and TiO${}_{x}$ [66].

In memristive devices that employ BFO as the switching layer, the VCM can be classified into two subcategories: interface resistive switching (VCM-interface RS) and filament resistive switching (VCM-filament RS). VCM-interface RS is based on the interaction between the BFO thin film and the electrode material. The applied voltage in this mechanism causes changes in the charge distribution at the interface between the BFO thin film and the electrode material, resulting in the formation of a conductive pathway established through the migration of Vo${}^{\cdot \cdot }$. Reversal of the applied voltage leads to the redistribution of the Vo${}^{\cdot \cdot }$ and the disappearance of the conductive pathway. VCM-filament RS, on the other hand, occurs as a result of the formation and subsequent rupture of conductive filaments within the BFO thin film. The formation of these filaments is initiated by an applied voltage, which leads to a change in the charge carrier concentration in the thin film and the creation of a conductive pathway. This pathway is established through the migration of Vo${}^{\cdot \cdot }$, serving as electron donors in the BFO thin film. Reversal of the applied voltage triggers the recombination of Vo${}^{\cdot \cdot }$, causing the conductive pathway to disappear and resulting in the rupture of the conductive filaments.

The physical mechanism of RS memory in a BFO memristive device was further investigated by You et al. [11] through the modeling of a modifiable Schottky barrier height, as shown in Figure 5b. The RS effect was demonstrated to be attributed to the adjustment of the Schottky barrier height at the BFO/Pt interface, which is induced by the application of an electric field. This leads to the migration of Vo${}^{\cdot \cdot }$ in the BFO functional layer, which serves as mobile donors. Simultaneously, Ti${}^{4+}$ ions act as fixed donors in the layer. The results highlight the importance of adjusting the Schottky barrier height for understanding the underlying mechanism of RS memory in BFO-based devices. As shown in Figure 5d, Chen et al. [62] concluded that the resistance switching observed in BFO-based memristive devices is a result of a bulk effect rather than being driven by interface effects. The resistance transition occurs at lower voltages than the coercive voltage and is unrelated to ferroelectric polarization. A tentative explanation for the sequence of events between the initial state, LRS, and HRS is proposed. The central mechanism behind the unipolar resistance switching in BFO is attributed to the presence of Vo${}^{\cdot \cdot }$, which exhibits relative ease of movement and the necessary conditions for the formation of conducting pathways.

In conclusion, the physical mechanisms of the RS effect in BFO-based memristive devices need extensive exploration. This review has aimed to provide a comprehensive overview of the current understanding of the physical mechanisms responsible for RS in BFO-based memristive devices. However, the complexity of the RS phenomena in BFO-based memristive devices still requires deeper insight and future research.

## 5. Resistive Switching Properties and Applications

### 5.1. RS Properties of BFO Devices

In the past decade, significant developments in the improvement of perovskite memristive devices based on BFO are performed, and the RS properties such as high on/off ratio, excellent retention and endurance, fast switching speed, and low energy consumption are attainable. Table 2 summarizes the RS properties of representative BFO-based memristive devices in the past decade. Table 2 comparably illustrates the data on the device structures, RS behaviors, RS mechanisms with their respective types, on/off ratio, set voltages (V${}_{set}$), reset voltages (V${}_{reset}$), endurance, and retention. However, these high-performance parameters have been achieved separately in different BFO-based memristive devices. Therefore, considerable effort is required to further improve the overall performance of memristive devices and promote the development and application of BFO in future high-density storage and neuromorphic computing.

As shown in Table 2, the device structures range of BFO-based memristive devices can be constructed by a single layer or multilayer structure integrated with distinct electrodes and buffer layers. These structural variations can influence the RS reliability, physical scalability, and RS mechanisms. The voltage range for V${}_{set}$ and V${}_{reset}$ typically varies between a positive bias of 0.4 to 11 V and a negative bias of −0.4 to −11 V, respectively. V${}_{set}$ and V${}_{reset}$ indicate the range of writing bias for individual BFO-based RS cells. The voltage magnitude depends on the thickness of the film, electrode material, and current compliance applied. The writing current values in LRS/HRS are highly dependent on the values chosen for recording them. Instead of LRS/HRS current values in specific conditions, the reported on/off ratio is summarized. The on/off ratio varies from 40 to 10${}^{5}$ with higher values indicating better memory performance. Endurance, the number of times the device can switch between resistance states without degradation, ranges from 50 to 10${}^{4}$ cycles, with higher values indicating better memory performance. Retention, the time that the device can maintain its resistance state without significant change, ranges from 10${}^{3}$ s to 10${}^{6}$ s, with higher values indicating better memory performance.

It is noteworthy that the RS devices based on BFO thin films prepared by different fabrication techniques could lead to the same physical switching mechanism but different dynamical switching properties. For example, concerning the fabrication techniques as illustrated in Figure 2 in Section 2 and lattice structures in Table 1 in Section 3, the RS devices based on Pt/BFO (250)/LNO (200 nm)/Si [25] and Au/BFO (600 nm)/Pt/Ti/SiO${}_{2}$/Si [17] with rhombohedral BFO thin films are prepared by the sol–gel method and PLD, respectively. Both RS devices possess the bipolar RS behavior originating from a VCM-based barrier switching mechanism but with around one order of magnitude difference in programming voltages V${}_{set}$ and V${}_{reset}$. Moreover, the RS devices based on BFO thin films with different lattice structures could result in different switching mechanisms but possess comparable RS properties. For example, the RS devices based on SRO/B(Ca)FO (300 nm)/SRO/STO [5] with tetragonal Ca${}^{2+}$ doped BFO thin films are fabricated by PLD, and their bipolar RS behavior is related to ferroelectricity-induced PN junction switching, which is different from the VCM induced RS found in the aforementioned device based on Au/BFO (600 nm)/Pt/Ti/SiO${}_{2}$/Si [17]. However, both of them are sharing similar RS properties as illustrated in Table 2. Based on the comparison, it is clear that there is no BFO memristive device to exhibit optimal RS behaviors. Different structures may have varying advantages and disadvantages depending on their application requirements. There is a trade-off between voltage magnitude and memory performance, with lower voltages reducing power consumption and device degradation but also lowering the on/off ratio and endurance. Device miniaturization provides higher density and possibly faster switching speed but in general a lower on/off ratio, retention, and endurance. Thus, it is important to carefully consider the optimization of fabrication processes, crystal structures, RS mechanisms, and other factors in order to enhance the performance of BFO-based memristive devices.

### 5.2. Applications Scenarios

**Figure 6:**
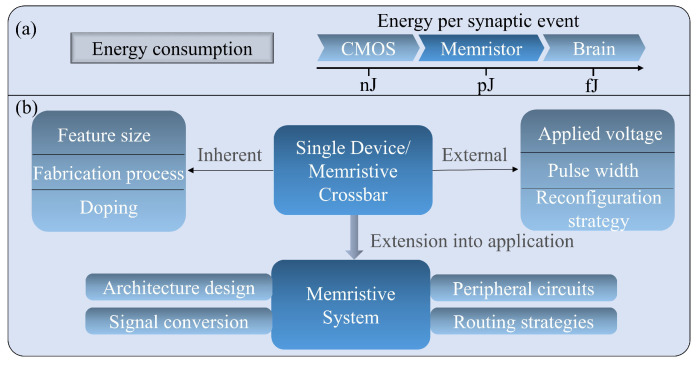
(**a**) Energy per synaptic event among CMOS, memristive device, and the brain; (**b**) Factors contributing to the energy consumption of single memristive devices and memristive systems.

Low energy cost is pursued in today’s applications, and the energy consumption is one of the most important concerns of a memristive system, especially in artificial neural network applications. As shown in Figure 6a, most artificial synaptic devices based on complementary metal-oxide semiconductor (CMOS) technology typically operate at ^~^ nJ per synaptic event level [67,68,69], and only a few studies reach several tens of pJ [70,71]. By exploiting memristive devices, it is much easier to emulate an artificial synaptic device with an energy cost of few pJ per synaptic event level [72,73] and can even approach hundreds of fJ [74,75], which is much closer to the energy cost of the human brain, i.e., 10 fJ per synaptic event, in comparison to CMOS approaches [76]. While memristive devices are well suited for the use of artificial synapses in brain-inspired information processing systems in neuromorphic computing, it is essential to study the energy consumption of memristive devices and suppress their energy cost to the lowest possible value. In order to evaluate the energy cost of any memristive devices in a valid manner, various factors as shown in Figure 6b shall be considered.

The energy consumption of a single memristive device is determined by different aspects, especially by the inherent physical switching mechanisms and the modulation signals applied externally, as shown in Figure 6b. The inherent physical switching mechanism is the in-depth underlying factor of energy consumption of memristive devices, e.g., the fabrication technique, doping effect, feature size, etc. In VCM interface-based memristive devices, energy consumption can be reduced by decreasing the area size of the top electrode [77]. With decreasing the TE area size, the capture/de-capture ions at the TE/oxide layer interface are reduced, which leads to an increase in the Schottky barrier height and reduces the current at the same testing bias. In memristive devices based on VCM filament switching or FTJs, the energy consumption is not influenced by the TE size; however, it can be reduced by increasing the thickness of the oxide films under the same testing bias. The thickness of the oxide film directly affects the complexity of the defect boundaries and influences the evolution/distribution of the conductive filaments. Hence, the resistance of the device in both HRS/LRS is affected [78]. Furthermore, as reported, the RS process in FTJs, which can reach ^~^ nA, consumes much less energy in terms of current than the comparison VCM [79]. Additionally, the doping effect has been demonstrated to significantly influence the energy consumption of memristive devices based on various RS mechanisms [80,81,82]. For example, Tan et al. [83] demonstrated that the introduction of nickel doping in HfO${}_{x}$ can reduce the energy consumption and enhance the multilevel resistance behavior under the same pulse operation.

In addition, the energy consumption of memristive devices is further influenced by the external modulation signal, i.e., the applied voltage with corresponding pulse widths, and the reconfiguration strategy, i.e., writing/reading schemes for operating memristive crossbar arrays. The high RS speed is one of the essential criteria for low energy consumption. The memristive devices can be switched rapidly in a few nanoseconds with only a few volts of electrical excitation. It is beneficial to reduce the energy consumption by minimizing the pulse width while ensuring the proper operation of the device. However, in order to obtain high switching speeds, a high operation voltage is generally required. It is known as the voltage–time dilemma when the pulse width exponentially grows as the pulse height linearly decreases, and this is a bottleneck in energy consumption reduction [84].

Regarding memristive devices based on BFO thin films, various combinations of operating voltage, current, and pulse width can be observed depending on the fabrication technique or RS mechanism. Typical observed operational voltages are confined to an interval between 0.5 and 15 V [5,7,10,19,21,27]. Conversely, the current range encompasses a broad spectrum, from ^~^ μA to ^~^ mA. Additionally, the RS time of memristive devices is in the ^~^ μs to ^~^ ns range. Note that in each memristive device, the energy consumption per operation can be different by more than several orders of magnitude due to the aforementioned voltage–time dilemma, i.e., the RS pulse width decreases exponentially while linearly increasing the bias amplitude [85]. However, the state-of-the-art works on the comparison of energy consumption among memristive devices [58,86,87] only used the reported pulse amplitudes and pulse widths in their typical working region without considering the extreme cases, which is invalid. One valid evaluation of the energy consumption of memristive devices shall be taken under extreme cases, i.e., the RS data recorded by applying the shortest possible pulse width. RS with the lowest possible pulse amplitude or current is not suggested. Memristive devices with no abrupt switching behavior have no deterministic threshold RS bias, which can complicate the definition of criteria for energy evaluation. Moreover, in order to take the stochastic variability of the memristive device into account, i.e., device-to-device (D2D) and cycle-to-cycle (C2C) variations, the mean value averaged by RS data recorded from a certain amount of SET/RESET cycles per device and a certain amount of device provides more accurate energy estimation of one memristive device. Unfortunately, such results are rarely available in today’s publications regarding memristive technology.

The energy consumption of a memristive computing system is not only determined by the energy cost of a single device but also by system-level factors, such as architecture design, signal conversion, peripheral circuitry, and routing strategies, as shown in Figure 6b.

(1) System architecture: The in-memory computing enabled by memristive devices could realize a substantial reduction in energy consumption due to their ability to data process in memory itself [88], which is impossible in conventional von Neumann architecture, e.g., SpiNNaker [89], Neurogrid [90], where their system energy consumption is majorly costed by memory assessment: the so-called memory wall bottleneck.

(2) Signal conversion: The memristive devices, in contrast to conventional CMOS devices, have the ability to maintain multiple states instead of being limited to binary states, i.e., analog switching behavior. The frequency and magnitude of signal conversion have a significant impact on the energy consumption of a system. The interconnection of multiple memristive crossbar arrays typically employs digital signaling, which introduces an additional layer of digital-to-analog conversion (DAC) and analog-to-digital conversion (ADC). Liu et al. [91] introduced the concept of a mixed-signal interconnect network (M-Net) for transferring data among memristive crossbar arrays in an analog form with the aim of minimizing the need for digital-to-analog signal conversion, thereby reducing the energy consumption of the system.

(3) Peripheral circuits: The memristive crossbar arrays of an in-memory computing system can be precisely controlled through the utilization of peripheral circuits, which primarily comprise ADCs/DACs, decoding logic, drivers, multiplexers, and shift-and-add circuitry, among others. It is noteworthy that ADCs/DACs consume the most energy among these components. Consequently, optimizing the circuit by replacing high-power consumption devices with alternatives that maintain performance is a viable approach. For peripheral circuit design of the neural network, replacing the ADCs/DACs with “digital-like” circuits such as D-flip flops, integrated-and-fire circuits, and counters can improve both energy consumption and latency [92,93].

(4) Routing strategies: The routing strategies define the interconnection rules among multiple memristive crossbar arrays, i.e., processing cores, in one memristive computing system. A power-optimized routing strategy that minimizes long-distance communication can be selected to reduce the energy consumption. Approaches commonly used include mesh routing, which offers high flexibility but consumes substantial resources, or tree routing, which conserves latency and power but restricts the network types that can be supported [94]. In addition to single routing schemes, the combination of various routing strategies can be utilized to design an architecture that minimizes the system-level bandwidth requirements for communication spikes between neurons, enabling minimum-hop transmission or area routing to save energy. Moradi et al. [95] have demonstrated the effectiveness of this approach by utilizing multiple routing strategies combined with heterogeneous memory structures, resulting in a depressed energy consumption of 17 pJ per hop.

Moreover, the flexible materials/substrates [96] have drawn much attention for facilitating the diverse needs in daily human life in the Internet of Things era. Especially, some pioneering work has demonstrated prototypes of flexible perovskite memristive devices [97,98,99], e.g., BFO-based flexible memristive devices, in applications of neural networks. For instance, Sun et al. [100] deposited high-quality BFO on flexible mica by designing SrRu$O_{3}$/BaTi$O_{3}$ as a buffer layer and accordingly implemented a flexible BFO-based perovskite memristive device for neuromorphic computing. The flexible BFO memristive device exhibits voltage-tuned multilevel resistance states, which are stable up to 10${}^{3}$ bending cycles. Hence, a spike-time-dependent plasticity (STDP) with bending is realizable. Another multi-functional potential in BFO layers is attributed to their optical property, which can be added up with their RS behaviors. For instance, Zheng et al. [101] explored illumination-controlled conversion between RS and negative differential resistance (NDR) behaviors in BFO/ZnO films, i.e., a photo-induced NDR, which can be applied to electronic information and optical quantum computers. This work opens a new pathway for fabricating optoelectronics multi-functioning RS devices.

## 6. Summary and Outlook

This review provides a comprehensive overview of RS in BFO memristive devices. BFO is a multiferroic material with ferroelectric properties that make it an ideal candidate for non-volatile information storage media. The study examines the fabrication techniques and crystal structures of BFO memristive devices as well as the RS mechanisms of BFO memristive devices, and the RS properties of various BFO devices are also summarized. The applications of RS in BFO are also presented, discussing the potential for enhancing the material’s performance and reducing energy consumption. This review highlights the significant progress made in the field of RS in BFO memristive devices as well as the potential for future advancements. Further research is needed to fully understand the mechanisms of RS in BFO and to explore the potential of other multiferroic nanomaterials for non-volatile memory technologies. The development of more efficient and reliable fabrication techniques, as well as a deeper understanding of the crystal structure of BFO, could greatly enhance the performance of BFO memristive devices. Ultimately, the advancements in this field could lead to the development of faster, higher density, and lower energy-consuming non-volatile memory technologies with broad implications for the fields of computing and information storage. 

## Figures and Tables

**Table 1:** Crystal symmetry and microstructure of devices based on rhombohedral BFO, including morphologies, device structures, crystal types, ferroelectricity, and RS mechanisms in Ref. [7,17,25,53].

Morphologies	Device Structures	Crystal Types	Ferroelectricity	RS Mechanisms	References
Nano thinfilm	Pt/BFO/Pt/Ti/SiO${}_{2}$/Si	Polycrystalline	N	Barrier switching	Shuai et al., 2011
Nano thinfilm	Pt/BFO/LNO/Si	Polycrystalline	Y	Barrier switching	Chen et al., 2012
Nano island	Pt/BFO/NSTO	Single crystalline	Y	Ferroelectricity & Barrier switching	Jeon et al., 2016
Nano island	Pt/BFO/LSMO/LAO	Single crystalline	Y	Ferroelectricity	Chen et al., 2019

**Table 2:** RS properties of several BFO-based memristive devices, including device structures, RS behaviors, RS mechanisms with their respective types, on/off ratio, set voltages (V${}_{set}$), reset voltages (V${}_{reset}$), endurance, and retention in Ref. [5,14,17,19,20,27,58,62].

Nr.	Device Structures	RS Behaviors	RS Mechanisms	On/Off Ratio	V*_set_* (V)	V*_reset_* (V)	Endurance (#)	Retention (s)	References
1	SRO/B(Ca)FO/SRO/STO	Bipolar	P-N/N-P junction switching	500	−12	+12	-	1.3 × 10^6^	Yang et al., 2009
2	Pt/BFO/Pt/Ti/SiO_2_/Si	Unipolar	Filament switching	10^3^	+1.4	+3.3	50	-	Chen et al., 2010
3	Au/BFO/Pt/Ti/SiO_2_/Si	Bipolar	Barrier switching	631	+11	−11	10^4^	2.3 × 10^5^	Shuai et al., 2011
4	Pt/BFO/LNO/Si	Bipolar	Barrier switching	10^3^	+0.4	−0.4	-	-	Luo et al., 2012
5	Pt/BFO/SRO/STO	Bipolar	Ferro- electricity	750	+3	−3	-	10^3^	Hong et al., 2013
6	Pt/BFO/NSTO	Bipolar	N-P junction switching	10^5^	+3	−5	-	10^4^	Zhao et al., 2017
7	Pt/BFO/SRO/STO	Bipolar	Filament switching	10^3^	−3	+3	300	10^3^	Wang et al., 2020
8	Pt/BF(Mn)O/TiN/SiO_2_/Si	Bipolar	Filament switching	40	−0.17	+0.9	10^4^	10^4^	Zhao et al., 2023

## Data Availability

Data available upon request.
